# *BRAF* V600E mutation mediates FDG-methionine uptake mismatch in polymorphous low-grade neuroepithelial tumor of the young

**DOI:** 10.1186/s40478-020-01023-3

**Published:** 2020-08-18

**Authors:** Kensuke Tateishi, Naoki Ikegaya, Naoko Udaka, Jo Sasame, Takahiro Hayashi, Yohei Miyake, Tetsuhiko Okabe, Ryogo Minamimoto, Hidetoshi Murata, Daisuke Utsunomiya, Shoji Yamanaka, Tetsuya Yamamoto

**Affiliations:** 1 Department of Neurosurgery, Graduate School of Medicine, Yokohama City University, 3-9 Fukuura, Kanazawa, Yokohama, 2360004 Japan; 2 Department of Pathology, Yokohama City University Hospital, Yokohama, Japan; 3 Department of Radiology, Graduate School of Medicine, Yokohama City University, Yokohama, Japan; 4 Departmento of Radiology, Division of Nuclear Medicine, National Center for Global Health and Medicine, Tokyo, Japan

**Keywords:** PLNTY, *BRAF* V600E mutation, Methionine PET, LAT1

## Abstract

We present a case of a 14-year old boy with tumor-associated refractory epilepsy. Positron emission tomography imaging demonstrated a region with heterogeneous high ^11^C-methionine uptake and a region with homogenous low ^18^F-fluorodeoxyglucose uptake within the tumor. Histopathological and genomic analyses confirmed the tumor as *BRAF* V600E-mutated polymorphous low-grade neuroepithelial tumor of the young (PLNTY). Within the high-methionine-uptake region, we observed increased protein levels of L-type amino acid transporter 1 (LAT1), a major transporter of methionine; c-Myc; and constituents of the mitogen-activated protein kinase (MAPK) pathway. We also found that LAT1 expression was linked to the *BRAF* V600E mutation and subsequent activation of MAPK signaling and c-Myc. Pharmacological and genetic inhibition of the MAPK pathway suppressed c-Myc and LAT1 expression in *BRAF* V600E-mutated PLNTY and glioblastoma cells. The BRAF inhibitor dabrafenib moderately suppressed cell viability in PLNTY. Collectively, our results indicate that *BRAF* V600E mutation-activated MAPK signaling and downstream c-Myc induces specific metabolic alterations in PLNTY, and may represent an attractive target in the treatment of the disease.

**Electronic supplementary material:**

The online version of this article (10.1186/s40478-020-01023-3) contains supplementary material, which is available to authorized users.

## Introduction

Pediatric low-grade neuroepithelial tumors (P-LGNTs) encompass a group of central nervous system neoplasms that includes long-term epilepsy-associated tumors (LEATs), such as ganglioglioma and dysembryoplastic neuroepithelial tumor (DNT). P-LGNTs have different characteristics than their adult counterparts, and are commonly driven by genomic alterations in the Ras/mitogen-activated protein kinase (MAPK) pathway, such as mutations in *BRAF* and *NF*-*1* [23, 29]. Recent large-scale genomic studies and genome-wide methylation analyses allowed a thorough characterization of P-LGNTs [24], and cIMPACT-NOW (the Consortium to Inform Molecular and Practical Approaches to CNS Tumor Taxonomy) currently classifies P–LGNTs as distinct disease entities [4, 17]. In 2017, Huse et al. described ten cases of polymorphous low-grade neuroepithelial tumor of the young (PLNTY), which were histologically characterized by oligodendroglioma-like cellular components with intense CD34 immunopositivity. According to previous publications, PLNTYs are indolent tumors that generally exhibit a benign clinical course and harbor either a *BRAF V600E* mutation or *FGFR2*/*FGFR3* fusion [9]. Based on its histological and genomic profiles, cIMPACT-NOW Update 6 recommends PLNTY as a possible future classification for pediatric-type glial/glioneuronal tumors. However, because of their rare etiology, only a few PLNTYs have been described to date [3, 5, 9, 10, 16, 21, 27, 28], and it is unclear how genomic alterations promote the pathogenesis of the disease. Herein, we present a case of PLNTY with unique metabolic imaging features. Using positron emission tomography (PET), we found regions of heterogeneous high ^11^C-methionine uptake and homogenous low ^18^F-fluorodeoxyglucose (FDG) uptake within the tumor. Activation of the MAPK pathway, c-Myc, and expression of L-type amino acid transporter 1 (LAT1) were increased in the high-methionine-uptake area compared with the surrounding cortex (low-methionine-uptake). Glycolytic metabolites were expressed only weakly in tumor cells. Pharmacological and genetic inhibition of the MAPK pathway suppressed c-Myc and LAT1 and inhibited tumor cell viability, suggesting that MAPK signaling and downstream c-Myc activates methionine metabolism and inhibition of this pathway induces therapeutic vulnerability in PLNTY.

## Materials and methods

### Cell viability analysis

AM-38 and normal human astrocytes was purchased from JCRB Cell Bank and ScienCell Research Laboratories, respectively. Tumorsphere lines were cultured in serum-free neural stem cell medium, as previously described [31]. Normal human astrocytes were cultured with astrocyte medium (ScienCell). To assess cell viability, primary cultured cells were dissociated into single cells and seeded into 96-well plates at a density of 3000 cells/well. After 12 h, dabrafenib (Selleck) and trametinib (Selleck) were serially diluted and added to the wells. Cell viability was measured using the CellTiter-Glo (Promega) assay at day 3, and the results were indicated as % viability of the DMSO control.

### shRNA cell line generation

To knockdown *BRAF*, 293T cells were transfected with lentiviral vector packaging plasmid DNA containing 6 μg of Human *BRAF* shRNA (#1, TRCN0000381693; #2, TRCN0000196844; Sigma Aldrich), 3.5 μg of pHIV-GP, and 3.5 μg of pVSVg-Rev with Lipofectamine™ 3000 (Thermo Fisher Scientific). YMG62 and AM-38 cells were infected with lentivirus in polybrene (8 μg/mL) for 12 h. Two days later, the cells were selected with puromycin (0.6 μg/mL) for 2 days, and used for experiments. GIPZ non-silencing lentiviral shRNA Control (RHS4348, Horizon Discovery) was used as a non-silencing (NS) control.

### Immunohistochemistry

Tumor tissue specimens were fixed in 10% neutral buffered formalin and embedded in paraffin. Hematoxylin and eosin staining was performed using standard procedures. For immunohistochemical analysis, 5-µm-thick sections were deparaffinized, treated with 0.5% H_2_O_2_ in methanol, rehydrated, and heated for 20 min for antigen retrieval. After blocking with serum, tissue sections were incubated with primary antibodies against CD34 (Novus Biologicals), LAT1 (Cell Signaling Technology), phospho-MEK (Cell Signaling Technology), phospho-ERK (Bethyl Laboratories), and c-Myc (Cell Signaling Technology) at 4 °C overnight. The next day, sections were washed with PBS, incubated with biotinylated secondary antibody for 30 min at room temperature, and then incubated with ABC solution (PK-6101, PK-6102; Vector laboratories) for 30 min at room temperature. Finally, the sections were incubated with DAB (Dako) and counter-stained with hematoxylin.

### Western blotting

Cells were lysed in RIPA buffer (Sigma-Aldrich) with a cOmplete™, Mini, EDTA-free Protease Inhibitor Cocktail (Roche). Fifty micrograms of protein was separated by 10% SDS-PAGE gel and transferred to polyvinylidene difluoride membranes (Millipore) by electroblotting. After blocking with 1% or 5% nonfat dry milk in TBST (25 mM Tris [pH, 7.4], 137 mM NaCl, 0.5% Tween20), membranes were incubated at 4 °C overnight with primary antibodies. After washing and incubation with horseradish peroxidase-conjugated secondary antibodies (Cell Signaling Technology), blots were washed, and signals were visualized with chemiluminescent HRP substrate (Millipore). Primary antibodies against BRAF (Gene Tex), c-Myc (Cell Signaling Technology), GAPDH (Gene Tex), LAT1 (Cell Signaling Technology), phospho-MEK (Cell Signaling Technology), phospho-ERK (Bethyl Laboratories), and Vinculin (Novus Biologicals) were used for western blotting.

## Case presentation

**Fig. 1:**
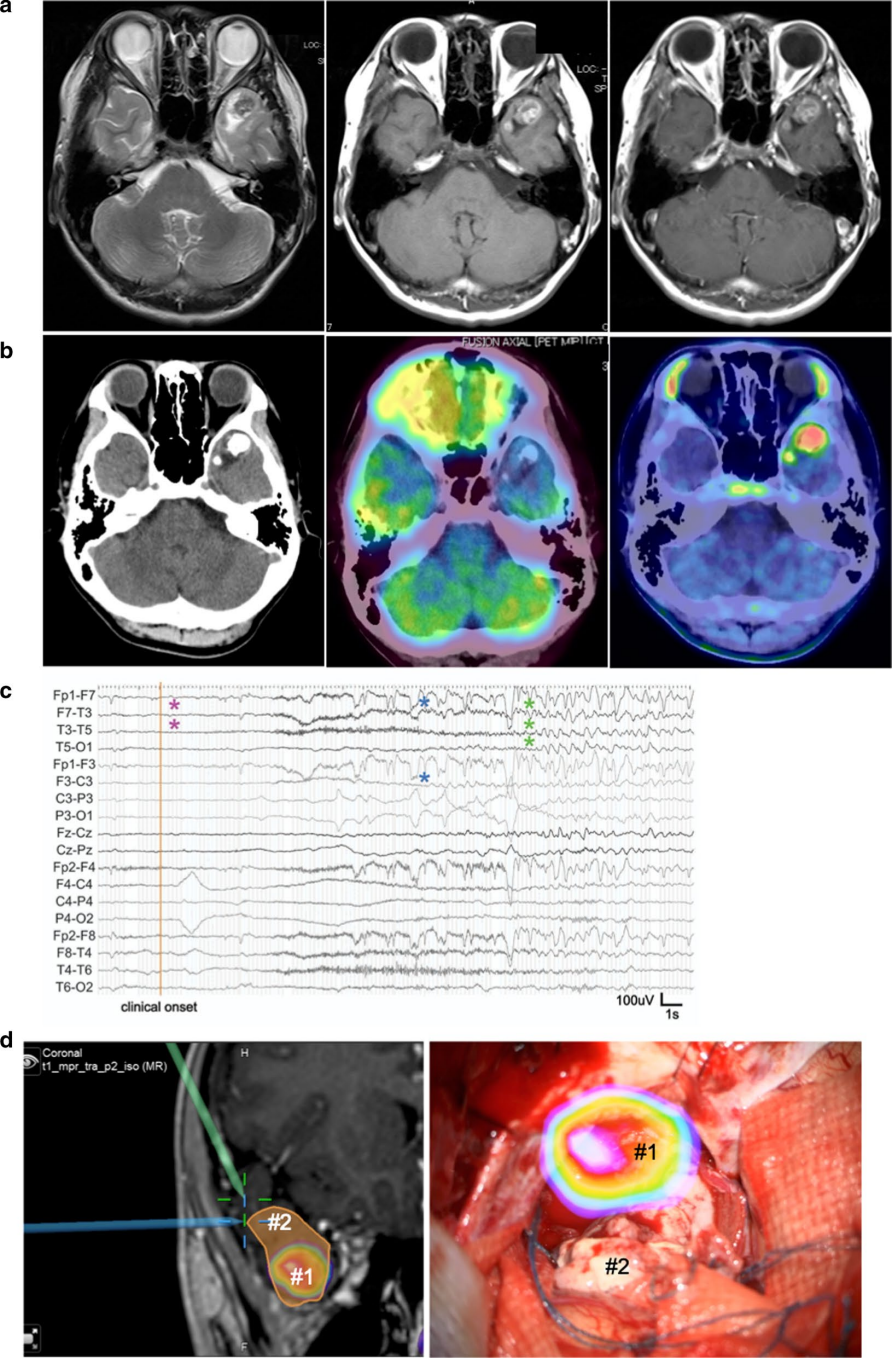
Characteristics of a patient with PLNTY. **a** T2-weighted (left), T1-weighted (middle), and contrast-enhanced (right) MR images. **b** Computed tomography (CT, left), ^18^F-fluorodeoxyglucose-PET/CT (middle), and ^11^C-methionine-PET/CT (right) images. **c** Video electroencephalography indicating ictal onset in the left temporal lobe, with spread to the contralateral temporal lobe. **d** PET/CT and MRI merged intraoperative navigation image (left) and surgical image (right) showing the high-methionine-uptake region (#1) and surrounding abnormal lesion (#2) on MRI

This study was performed in accordance with declaration of Helsinki and was approved by the Institutional Review Board (Yokohama City University [YCU, Yokohama, Japan], IRB numbers: A1711300006 and B190600002). Written informed consent was obtained from the patient and parents. A 14-year old boy presented with chronic medial temporal lobe epilepsy for a year. Magnetic resonance imaging (MRI) indicated hypointensity on T2-weighted images and hyperintensity on T1-weighted images, with a cystic component in the left temporal lobe. Contrast-enhanced MRI showed no significant enhancement in the lesion (Fig. 1a) while computed tomography revealed heavy calcification. FDG-PET showed lower FDG uptake in the tumor, while ^11^C-methionine-PET demonstrated increased methionine uptake in the same lesion (SUVmax = 3.9, tumor/normal tissue ratio = 2.9; Fig. 1b). Video-electroencephalographic (EEG) monitoring indicated ictal onset in the left temporal lobe with subsequent spread to the contralateral temporal lobe (Fig. 1c). We speculated that this abnormal lesion was a LEAT. Since we considered this tumor to be completely resectable, the patient underwent craniotomy and resection of the neoplasm, including the high-methionine-uptake region (Fig. 1d). To achieve epileptic control, electrocorticography was performed intraoperatively. After removal of the high-methionine-uptake and T2 hyperintense lesions, the surrounding tissue was resected until interictal epileptiform discharge could no longer be detected by electrocorticography. The patient became epilepsy-free after lesion removal, and MRI indicated complete remission 16 months after the surgery.

**Fig. 2:**
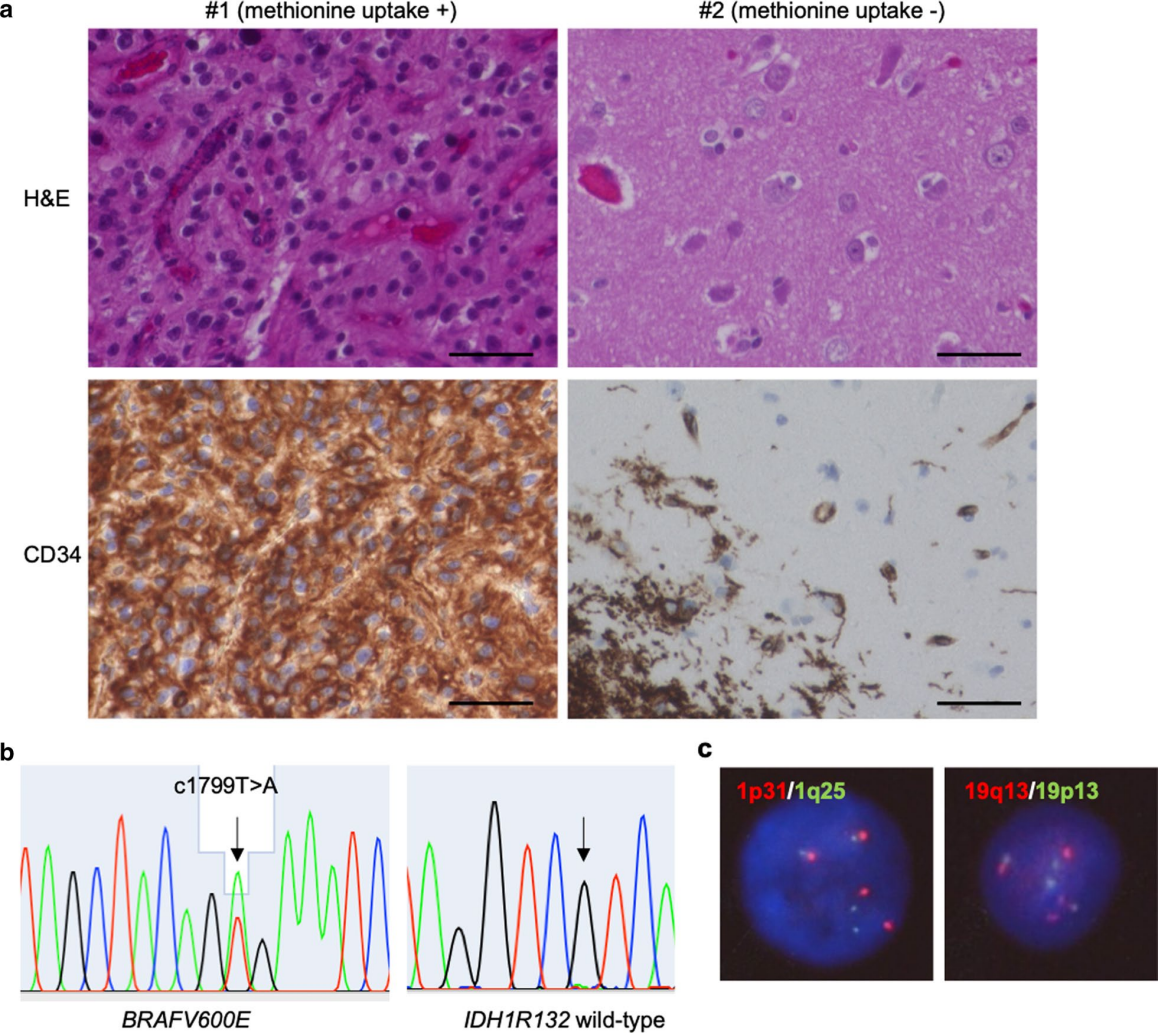
Histopathologic and genomic features of a patient with PLNTY. **a** Hematoxylin and eosin (H&E) staining (top) and CD34 immunohistochemistry (bottom) in the high-methionine-uptake (#1) and low-methionine-uptake (#2) region within tumor tissue. Bars, 50 μm. **b** Sanger sequencing for detection of *BRAF* V600E (arrow, left) and *IDH1* R132H (arrow, right) mutations. **c** Fluorescence in situ hybridization for detection of 1p31/1q25 (left) and 19q13/19p13 (right) chromosomal deletions

Tissue samples of the high-methionine-uptake region (#1) and surrounding cortex (low methionine uptake, #2) were collected. Hematoxylin and eosin staining indicated diffusely infiltrating growth patterns and presence of oligodendroglia-like cellular components (Fig. 2a). Astrocytic and high-grade features were absent, with a Ki-67 index of less than 2%. Chicken wire-like branching capillaries and microcalcification were also found in region #1. Despite lower cellularity, oligodendroglia-like cells were present in the surrounding tissue. Immunohistochemistry revealed extensive CD34 expression and peripherally associated ramified neural elements in the tumor cells (Fig. 2a). Targeted DNA sequencing identified a *BRAF* V600E mutation in the tumor, without recurrent mutations in *IDH1, IDH2*, *TERT* promoter, *FGFR1*, *H3F3A*, or *HIST3H1B* (Fig. 2b). Chromosome 1p/19q co-deletion was absent (Fig. 2c). The above histological and genetic features fulfilled the diagnostic criteria for PLNTY.

**Fig. 3:**
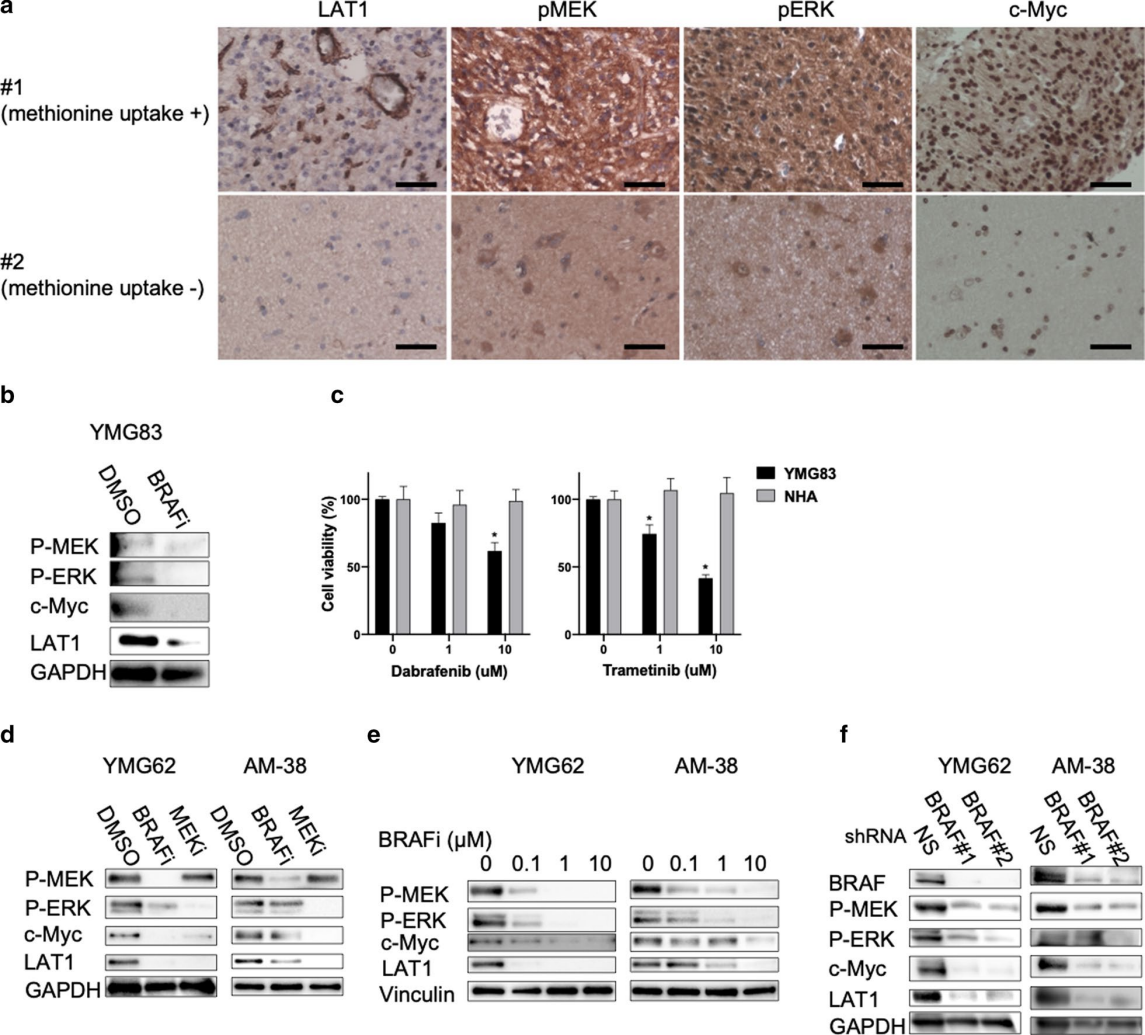
Activating the MAPK pathway induces LAT1 expression in a patient with PLNTY. **a** Immunohistochemistry of indicated proteins in the high-methionine-uptake (#1) and low-methionine-uptake (#2) regions within tumor tissue. Bars, 50 μm. **b** Western blot analysis of phospho-MEK, phospho-ERK, c-Myc, and LAT1 proteins in YMG83 (PLNTY, left) cells treated with DMSO and 10 μM BRAF inhibitor (BRAFi, dabrafenib) for 12 h. GAPDH, loading control. **c** Relative cell viability of dabrafenib-treated (left) and trametinib-treated (right) YMG83 cells and immortalized normal human astrocytes (NHA). *P < 0.05, DMSO versus dabrafenib (left) and trametinib (right). **d** Western blot analysis for indicated proteins in YMG62 (epithelioid glioblastoma, left) and AM-38 (glioblastoma, right) cells treated with DMSO, 10 μM BRAF inhibitor (BRAFi, dabrafenib), and 10 μM MEK inhibitor (MEKi, trametinib) for 24 h. GAPDH, loading control. **e** Western blot analysis of BRAF, phospho-MEK, phospho-ERK, c-Myc, and LAT1 proteins in YMG62 (left) and AM-38 (right) cells treated with DMSO and dabrafenib at indicated concentrations. Vinculin, loading control. **f** Western blot analysis for indicated proteins in *non*-*silencing*- (*NS*) and *BRAF*- (#1 and #2) transduced YMG62 and AM38 cells. GAPDH, loading control

To assess the mechanisms underlying the methionine-FDG uptake mismatch indicated by PET, we compared the expression of LAT1, glucose transporter 1 (GLUT-1), and hexokinase-2 (HK-2) between tissue regions #1 and #2. Notably, LAT1, which is a major methionine transporter, was more highly expressed in #1 than in #2 (Fig. 3a). In contrast, GLUT-1 and HK-2, which is correlated with FDG uptake, and lactate dehydrogenase A (LDHA) expression were weak in either region (Additional file 1: Fig. S1). LAT1 expression is mediated by c-Myc activation and *BRAF* V600E mutation activates the MAPK pathway and downstream c-Myc [8, 32, 33]. Therefore, we hypothesized that *BRAF* V600E mutation promotes LAT1 expression through MAPK signaling and consequent c-Myc activation in PLNTY. Levels of phospho-MEK, phospho-ERK, and c-Myc were higher in tissue region #1 than in #2 (Fig. 3a), suggesting activation of the MAPK pathway and c-Myc within the high-methionine-uptake lesion. To verify whether the *BRAF* V600E mutation can induce the expression of LAT1, we exposed primary cultured YMG83 PLNTY cells to a BRAF inhibitor (dabrafenib). As expected, the expression of phospho-MEK, phospho-ERK, c-Myc, and LAT1 was suppressed after dabrafenib treatment in YMG83 cells (Fig. 3b). Notably, BRAF inhibitor (dabrafenib)-treated YMG83 cells had lower cell viability compared to normal human astrocytes (Fig. 3c). To confirm the reproducibility of these molecular features, we used patient-derived YMG62 cells (epithelioid glioblastoma with the *BRAF* V600E mutation), which exhibited high ^11^C-methionine uptake by PET imaging (Additional file 1: Fig. S2), and AM-38 glioblastoma cells (*BRAF* V600E mutant). We found that dabrafenib and a MEK inhibitor (trametinib) inhibited the expression of proteins in the MAPK pathway as well as c-Myc and LAT1 (Fig. 3d and 3e). Similarly, *BRAF* knockdown suppressed the expression of proteins in the MAPK pathway as well as c-Myc and LAT1 (Fig. 3f). Collectively, these findings indicated that activation of the MAPK pathway by the *BRAF* V600E mutation deregulates c-Myc and promotes LAT1 expression. This oncogenic signaling pathway increases methionine metabolism and tumor maintenance in PLNTY.

## Discussion

Thirty cases of PLNTY have been described to date, with the first ten reported by Huse et al. in 2017 [3, 5, 9, 10, 16, 21, 27, 28]; *BRAF* V600E mutation was seen in 14 of the patients and *BRAF* fusion in 1 patient. These *BRAF* alterations were mutually exclusive with other genomic events, including *FGFR3*-*TACC3* fusion, *FGFR3* amplification, *FGFR2*-*CTNNA3* fusion, *FGFR2*-*INA* fusion, *FGFR2*- *KIAA1598* fusion, *FGFR2* rearrangement, and *NTRK2* disruption, suggesting that the vast majority of PLNTYs are induced by *BRAF* mutation or *FGFR* fusion and subsequent MAPK activation. Therefore, targeting MAPK signaling may become a potential therapeutic strategy in PLNTY. Indeed, *BRAF* V600E-mutated PLNTY cells were relatively vulnerable to dabrafenib and trametinib in the present study. Thus, targeted molecular therapy for the MAPK pathway may be particularly useful in PLNTY located in surgically unresectable regions. In addition, Koh et al. reported that the *BRAF* V600E mutation contributes to the intrinsic epileptogenicity in pediatric brain tumors, and that inhibition of BRAF suppressed epileptic seizures [14]. Thus, BRAF/MEK inhibitors could exert anti-epileptic as well as anti-tumor effects in PLNTY.

PET imaging revealed a region with increased methionine uptake and low FDG uptake within tumor tissue in our patient. Consistent with this finding, previous case reports demonstrated increased methionine uptake but only mild FDG uptake in patients with *BRAF* V600E-mutated PLNTY [5, 16]. Thus, excessive methionine uptake and low FDG uptake may be imaging features specific to PLNTY. A preclinical study has demonstrated that high uptake of ^18^F-FDG was correlated with increased Glut-1 and HK-2 expression in human cancers [19]. Although the diagnostic accuracy is insufficient, FDG-PET imaging is useful to differentiate high-grade from low-grade gliomas [1]. In the present case, low FDG uptake and weak expression of Glut-1, HK-2, and LDHA were observed in tumor tissue, suggesting low glycolytic activity in PLNTY. On the other hand, due to a high signal-to-noise ratio, ^11^C-methionine PET imaging is practical for brain tumors [12, 34]. Several PET imaging studies have demonstrated that methionine uptake was higher in high-grade adult gliomas than in lower-grade gliomas [7, 30]. In epileptogenic brain tumors, however, all gangliogliomas and 37–57% of DNT had increased methionine uptake, although these tumors are classified as WHO grade I [20, 22], implying that methionine uptake may be irrespective of tumor grade in LEATs.

Previous studies have reported that methionine uptake was correlated with LAT1 in gliomas [13, 18]. LAT1 plays a major role in the transport of neutral essential amino acids, including methionine, and is driven by several cancer-related genes such as *MYC* [25]. It has been demonstrated that c-Myc, which is partly mediated by the MAPK pathway, regulates LAT1 expression and MEK inhibitor suppresses *LAT1 (SLC7A5)* transcription [6, 8], thereby indicating a role of the MAPK pathway and c-Myc in the regulation of LAT1. Since RAS/MAPK pathway-associated genomic alterations are common in LEATs [24] and that the *BRAF* V600E mutation has been identified in 20–60% and 30% of gangliogliomas and DNTs, respectively [2, 26], there is a possibility that the *BRAF* V600E mutation and MAPK pathway-related genomic alterations may activate methionine metabolism in LEATs. To investigate this hypothesis, we evaluated the protein expression of LAT1 and the molecules that are involved in the MAPK pathway. As expected, levels of phospho-MEK, phospho-ERK, c-Myc, and LAT1 were higher in the high-methionine-uptake area than in the low-methionine-uptake area. We also found that genetic and/or pharmacological BRAF inhibition suppressed MAPK pathway activation and attenuated LAT1 expression in *BRAF* V600E-mutated-PLNTY cells and -glioblastoma cell lines. These findings support the hypothesis that the *BRAF* V600E mutation may upregulate LAT1 and methionine metabolism through c-Myc activation for cell survival. In addition to LAT1, methionine uptake was correlated with microvascular density (MVD) in gliomas [15]. PLNTYs are considered benign brain neoplasms (proposed as WHO grade I); however, in the present case, a chicken wire-like MVD, which is one of the histopathological characteristics of oligodendroglioma, was also observed in the high-methionine-uptake tissue region. Intriguingly, methionine uptake has been reported to be relatively higher in oligodendrogliomas than in astrocytomas [11]. Thus, PLNTY, which has an oligodendroglioma-like microvascular structure, might show unique metabolic imaging features. Further studies are warranted to validate this hypothesis. Nonetheless, our data indicated that the *BRAF* V600E mutation induced MAPK pathway activation and downstream c-Myc promoted LAT1 expression and methionine metabolism with little effect on glycolytic pathway activation. These findings may explain the unique metabolic imaging features of FDG-methionine mismatch in PLNTY.

## Supplementary information

**Additional file 1: Figure S1.** Low glycolysis activation in a patient with PLNTY. Immunohistochemistry for glucose transporter 1, hexokinase 2, and lactate dehydrogenase A in the high-methionine-uptake (#1, upper) and low-methionine-uptake (#2, lower) region within tumor tissue. A. Bars, 50 μm. **Figure S2.** Images of the patient’s glioblastoma with the BRAF V600E mutation. Contrast-enhanced magnetic resonance (left) and 11C-methionine positron emission tomography (right) images of the YMG62 patient.

## References

[1] Borbely K, Nyary I, Toth M, Ericson K, Gulyas B (2006). Optimization of semi-quantification in metabolic PET studies with 18F-fluorodeoxyglucose and 11C-methionine in the determination of malignancy of gliomas. J Neurol Sci.

[2] Chappe C, Padovani L, Scavarda D, Forest F, Nanni-Metellus I, Loundou A, Mercurio S, Fina F, Lena G, Colin C (2013). Dysembryoplastic neuroepithelial tumors share with pleomorphic xanthoastrocytomas and gangliogliomas BRAF(V600E) mutation and expression. Brain Pathol.

[3] Chen Y, Tian T, Guo X, Zhang F, Fan M, Jin H, Liu D (2020). Polymorphous low-grade neuroepithelial tumor of the young: case report and review focus on the radiological features and genetic alterations. BMC Neurol.

[4] Ellison DW, Hawkins C, Jones DTW, Onar-Thomas A, Pfister SM, Reifenberger G, Louis DN (2019). cIMPACT-NOW update 4: diffuse gliomas characterized by MYB, MYBL1, or FGFR1 alterations or BRAF(V600E) mutation. Acta Neuropathol.

[5] Gupta VR, Giller C, Kolhe R, Forseen SE, Sharma S (2019). Polymorphous low-grade neuroepithelial tumor of the young: a case report with genomic findings. World Neurosurg.

[6] Hafliger P, Graff J, Rubin M, Stooss A, Dettmer MS, Altmann KH, Gertsch J, Charles RP (2018). The LAT1 inhibitor JPH203 reduces growth of thyroid carcinoma in a fully immunocompetent mouse model. J Exp Clin Cancer Res.

[7] Hatakeyama T, Kawai N, Nishiyama Y, Yamamoto Y, Sasakawa Y, Ichikawa T, Tamiya T (2008). 11C-methionine (MET) and 18F-fluorothymidine (FLT) PET in patients with newly diagnosed glioma. Eur J Nucl Med Mol Imaging.

[8] Hayashi K, Jutabha P, Endou H, Anzai N (2012). c-Myc is crucial for the expression of LAT1 in MIA Paca-2 human pancreatic cancer cells. Oncol Rep.

[9] Huse JT, Snuderl M, Jones DT, Brathwaite CD, Altman N, Lavi E, Saffery R, Sexton-Oates A, Blumcke I, Capper D (2017). Polymorphous low-grade neuroepithelial tumor of the young (PLNTY): an epileptogenic neoplasm with oligodendroglioma-like components, aberrant CD34 expression, and genetic alterations involving the MAP kinase pathway. Acta Neuropathol.

[10] Johnson DR, Giannini C, Jenkins RB, Kim DK, Kaufmann TJ (2019). Plenty of calcification: imaging characterization of polymorphous low-grade neuroepithelial tumor of the young. Neuroradiology.

[11] Kato T, Shinoda J, Oka N, Miwa K, Nakayama N, Yano H, Maruyama T, Muragaki Y, Iwama T (2008). Analysis of 11C-methionine uptake in low-grade gliomas and correlation with proliferative activity. AJNR Am J Neuroradiol.

[12] Katsanos AH, Alexiou GA, Fotopoulos AD, Jabbour P, Kyritsis AP, Sioka C (2019). Performance of 18F-FDG, 11C-methionine, and 18F-FET PET for glioma grading: a meta-analysis. Clin Nucl Med.

[13] Kobayashi K, Ohnishi A, Promsuk J, Shimizu S, Kanai Y, Shiokawa Y, Nagane M (2008) Enhanced tumor growth elicited by L-type amino acid transporter 1 in human malignant glioma cells. Neurosurgery 62:493–503; discussion 503–494 10.1227/01.neu.0000316018.51292.19 10.1227/01.neu.0000316018.51292.19 18382329

[14] Koh HY, Kim SH, Jang J, Kim H, Han S, Lim JS, Son G, Choi J, Park BO, Heo WD (2018). BRAF somatic mutation contributes to intrinsic epileptogenicity in pediatric brain tumors. Nat Med.

[15] Kracht LW, Friese M, Herholz K, Schroeder R, Bauer B, Jacobs A, Heiss WD (2003). Methyl-[11C]- l-methionine uptake as measured by positron emission tomography correlates to microvessel density in patients with glioma. Eur J Nucl Med Mol Imaging.

[16] Lelotte J, Duprez T, Raftopoulos C, Michotte A (2020). Polymorphous low-grade neuroepithelial tumor of the young: case report of a newly described histopathological entity. Acta Neurol Belg.

[17] Louis DN, Wesseling P, Aldape K, Brat DJ, Capper D, Cree IA, Eberhart C, Figarella-Branger D, Fouladi M, Fuller GNet al (2020) cIMPACT-NOW update 6: new entity and diagnostic principle recommendations of the cIMPACT-Utrecht meeting on future CNS tumor classification and grading. Brain Pathol. 10.1111/bpa.12832 10.1111/bpa.12832 PMC8018152 32307792

[18] Okubo S, Zhen HN, Kawai N, Nishiyama Y, Haba R, Tamiya T (2010). Correlation of L-methyl-11C-methionine (MET) uptake with L-type amino acid transporter 1 in human gliomas. J Neurooncol.

[19] Ong LC, Jin Y, Song IC, Yu S, Zhang K, Chow PK (2008). 2-[18F]-2-deoxy-d-glucose (FDG) uptake in human tumor cells is related to the expression of GLUT-1 and hexokinase II. Acta Radiol.

[20] Rheims S, Rubi S, Bouvard S, Bernard E, Streichenberger N, Guenot M, Le Bars D, Hammers A, Ryvlin P (2014). Accuracy of distinguishing between dysembryoplastic neuroepithelial tumors and other epileptogenic brain neoplasms with [(1)(1)C]methionine PET. Neuro Oncol.

[21] Riva G, Cima L, Villanova M, Ghimenton C, Sina S, Riccioni L, Munari G, Fassan M, Giangaspero F, Eccher A (2018). Low-grade neuroepithelial tumor: unusual presentation in an adult without history of seizures. Neuropathology.

[22] Rosenberg DS, Demarquay G, Jouvet A, Le Bars D, Streichenberger N, Sindou M, Kopp N, Mauguiere F, Ryvlin P (2005). [11C]-Methionine PET: dysembryoplastic neuroepithelial tumours compared with other epileptogenic brain neoplasms. J Neurol Neurosurg Psychiatry.

[23] Ryall S, Tabori U, Hawkins C (2020). Pediatric low-grade glioma in the era of molecular diagnostics. Acta Neuropathol Commun.

[24] Ryall S, Zapotocky M, Fukuoka K, Nobre L, Guerreiro Stucklin A, Bennett J, Siddaway R, Li C, Pajovic S, Arnoldo A (2020). Integrated molecular and clinical analysis of 1,000 pediatric low-grade gliomas. Cancer Cell.

[25] Salisbury TB, Arthur S (2018). The regulation and function of the l-type amino acid transporter 1 (LAT1) in cancer. Int J Mol Sci.

[26] Schindler G, Capper D, Meyer J, Janzarik W, Omran H, Herold-Mende C, Schmieder K, Wesseling P, Mawrin C, Hasselblatt M (2011). Analysis of BRAF V600E mutation in 1,320 nervous system tumors reveals high mutation frequencies in pleomorphic xanthoastrocytoma, ganglioglioma and extra-cerebellar pilocytic astrocytoma. Acta Neuropathol.

[27] Sumdani H, Shahbuddin Z, Harper G, Hamilton L (2019). Case report of rarely described polymorphous low-grade neuroepithelial tumor of the young and comparison with oligodendroglioma. World Neurosurg.

[28] Surrey LF, Jain P, Zhang B, Straka J, Zhao X, Harding BN, Resnick AC, Storm PB, Buccoliero AM, Genitori L (2019). Genomic analysis of dysembryoplastic neuroepithelial tumor spectrum reveals a diversity of molecular alterations dysregulating the MAPK and PI3K/mTOR pathways. J Neuropathol Exp Neurol.

[29] Tateishi K, Nakamura T, Yamamoto T (2019). Molecular genetics and therapeutic targets of pediatric low-grade gliomas. Brain Tumor Pathol.

[30] Tateishi K, Tateishi U, Nakanowatari S, Ohtake M, Minamimoto R, Suenaga J, Murata H, Kubota K, Inoue T, Kawahara N (2014). (62)Cu-diacetyl-bis (N(4)-methylthiosemicarbazone) PET in human gliomas: comparative study with [(18)F]fluorodeoxyglucose and L-methyl-[(11)C]methionine PET. AJNR Am J Neuroradiol.

[31] Wakimoto H, Kesari S, Farrell CJ, Curry WT, Zaupa C, Aghi M, Kuroda T, Stemmer-Rachamimov A, Shah K, Liu TC (2009). Human glioblastoma-derived cancer stem cells: establishment of invasive glioma models and treatment with oncolytic herpes simplex virus vectors. Cancer Res.

[32] Yue M, Jiang J, Gao P, Liu H, Qing G (2017). Oncogenic MYC activates a feedforward regulatory loop promoting essential amino acid metabolism and tumorigenesis. Cell Rep.

[33] Zhang W, Liu HT (2002). MAPK signal pathways in the regulation of cell proliferation in mammalian cells. Cell Res.

[34] Zhao C, Zhang Y, Wang J (2014). A meta-analysis on the diagnostic performance of (18)F-FDG and (11)C-methionine PET for differentiating brain tumors. AJNR Am J Neuroradiol.

