# Supplementary figures and images for: BRAF V600E mutation mediates FDG-methionine uptake mismatch in polymorphous low-grade neuroepithelial tumor of the young
Source: Acta Neuropathol Commun. 2020 Aug 18;8:139. doi: 10.1186/s40478-020-01023-3 (PMC7436956; doi:10.1186/s40478-020-01023-3)

Supplementary Figure 1

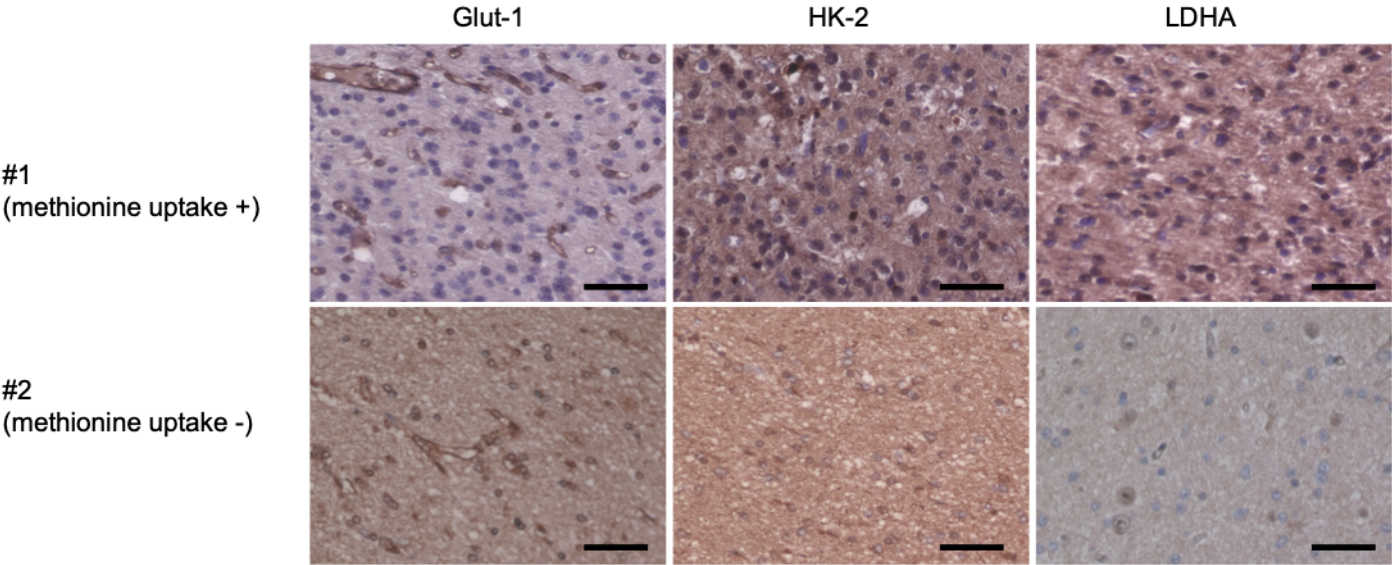

Supplementary Figure 2

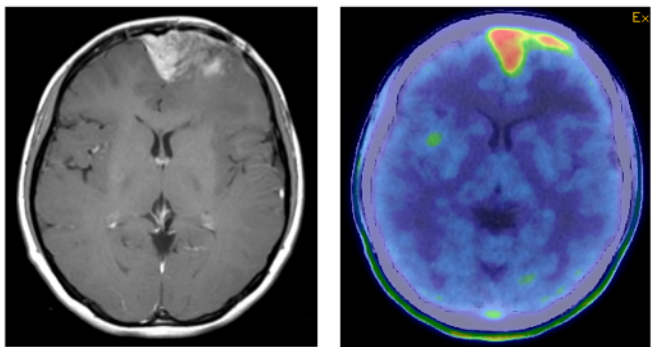

Supplement: Supplementary file 1 — Additional file 1: Figure S1. Low glycolysis activation in a patient with PLNTY. Immunohistochemistry for glucose transporter 1, hexokinase 2, and lactate dehydrogenase A in the high-methionine-uptake (#1, upper) and low-methionine-uptake (#2, lower) region within tumor tissue. A. Bars, 50 μm. Figure S2. Images of the patient’s glioblastoma with the BRAF V600E mutation. Contrast-enhanced magnetic resonance (left) and 11C-methionine positron emission tomography (right) images of the YMG62 patient. [file 40478_2020_1023_MOESM1_ESM.pdf]
